# How Pediatric Urology Fellowships in the United States are Funded

**DOI:** 10.3389/fped.2014.00119

**Published:** 2014-11-14

**Authors:** Brent Walter Snow, M. Chad Wallis

**Affiliations:** ^1^Division of Pediatric Urology, Primary Children’s Medical Center, University of Utah School of Medicine, Salt Lake City, UT, USA

**Keywords:** fellowship funding, pediatric urology, graduate medical education, fellowship training, revenue from pediatric urology fellows, pediatric urology fellowships

## Abstract

**Background:** We desired to discover how pediatric urology fellowship positions in the United States were funded.

**Methods:** Approved pediatric urology fellowship directors (25) were contacted by e-mail and asked how the 2 years of fellowship were funded at their institutions.

**Results:** The response rate of the e-mail questions was 100%. The clinical year of the fellowship was 100% hospital-funded in 88% of the 25 fellowships. The second, American board of urology (ABU)-required year was 100% hospital-funded in only 44% of the fellowships. Clinical funds generated by pediatric urology faculty provided funding for 24% of the fellows and institutes and grants funded 20% of the fellowship positions for the second year. Thirty-two percent of the fellowship positions have supplemental funding through charges generated from the fellow’s clinical activities in patient care.

**Conclusion:** All but three hospitals fund 100% of the clinical year of pediatric urology fellowship. Sources of funding for the second, ABU-required year vary widely among fellowship programs in the United States.

## Introduction

Urology residency positions in the United States have traditionally been funded through Medicare payments to hospitals that participate in residency training. The federal funding in hospitals for residency training has been capped since 1997, even though there are 200 more positions in traditional urology residencies since that time (1). Hospitals have the prerogative to distribute these funds through their Graduate Medical Education Office according to local needs and priorities.

Medicaid is the second largest source of funding despite the fact that there are no federal requirements for Medicaid programs to contribute to graduate medical education. It accounted for an estimated $3.78 billion dollars of funding for graduate medical education in 2009, compared to the $9.5 billion paid out by Medicare for direct and indirect funding (2). In 2005, 47 states contributed funding through their Medicaid programs, but by 2009 that number had dropped to 41 with an additional 9 states considering ending payments for graduate medical education because of budget concerns (3).

Pediatric training positions typically did not receive Medicare funding directly, especially if the training occurred in independent children’s hospitals where Medicare was not a large part of the insurance pool. Funding for these positions sometimes came from the Graduate Medical Education Office funds and other times through the School of Medicine or the children’s hospitals funds. In 1999, congress passed an authorization to fund pediatric residency training positions, which requires congressional renewal each year (4). This yearly renewal has left a cloud of uncertainty over new residency positions and has often been considered for budget cuts.

In the United States, the first accreditation council for graduate medical education (ACGME) accredited specialty within urology was pediatric urology. Pediatric urologists in the United States complete a urology residency after graduation from medical school. These programs are either 5 or 6 years depending on the program, but must include 1 year of training in general surgery. Interested urology residents will typically apply for fellowship positions the year prior to their final year of residency. These accredited 2-year fellowships began over two decades ago and this eventually led the American board of urology (ABU) to offer a Pediatric Subspecialty Certification Exam and pediatric subspecialty certification. The ACGME accreditation required 1 year of training, but the ABU-required 2 years of training to qualify for the pediatric subspecialty certification exam. This has led to a funding dilemma. The ACGME required clinical year funding is more straight forward; however, the second year required by the ABU can be either clinical or laboratory. Most of these positions are in children’s hospitals where Medicare Direct Graduate Medical Education funds may not be available (5), especially when the funds have been frozen for the last 15 years making institutions reluctant to create commitments to new pediatric urology fellowship positions. In addition, Medicaid funding for graduate medical education is non-existent in some states and tenuous in many others as has been discussed.

At our own freestanding children’s hospital, we have decided to offer a pediatric urology fellowship that requires residency review committee (RRC) approval. We sought information from program directors of already approved pediatric urology fellowship positions to learn how these positions were funded for the clinical year and the second, ABU-required year.

## Methods

The Society for Pediatric Urology website (www.spuonline.org) was queried for pediatric urology accredited fellowship positions and the program directors of each of these positions. The program directors of each of these positions were contacted by e-mail query. They were asked the following questions about their program’s funding:

1. How was the first clinical year funded at your institution?
2. How is the second “laboratory” year funded at your institution?
3. Are there clinical billings for the work that the pediatric urology fellows perform?

No external funding was received for this project and it was deemed exemption from IRB review as it did not involve human subjects.

## Results

There were 25 program directors or chairs who were contacted and all responded. Table 1 shows the first clinical year of funding, which may or may not be the first fellowship year. This shows 88% of the clinical years to be fully funded by the institution wherein the pediatric urology fellowship resides. One fellowship had a combination of 90% hospital and 10% clinical funding.

**Table 1:** **First clinical year of pediatric urology fellowship funding 25 approved pediatric urology fellowship training sites**.

Hospital only funding (100%)	22	88%
Clinical funding only	1	4%
Other	1	4%
Combination	1	4%

Table 2 shows funding for the second, ABU-required year. Only 44% of the time did the institution fund this year completely. Institutes or endowments funded this year in 20% of the programs and another 24% of the programs utilized clinical income from the faculty members to fund the additional fellowship year.

**Table 2:** **Second ABU-required year of pediatric urology fellowship funding 25 approved pediatric urology fellowship training sites**.

Hospital only funding (100%)	11	44%
Grants	2	8%
Institutes/endowments	5	20%
Combination	1	4%
Clinical income funding (100%)	6	24%

When asked if pediatric urology fellows had billings for their services during either of the fellowship training year, eight (32%) of the fellowships do submit bills for the patient care services provided by pediatric urology fellows. Sometimes, the billing was noted to be limited to ½–1 day a week and other fellowship directors did not specify any restrictions.

## Discussion

New residency programs since 1997 have suffered with the lack of government funding for these positions. Pediatric positions have often suffered to a greater degree without Medicare or Center for Medicare and Medicaid Services (CMS) based funding at children’s hospitals with few Medicare patients. Prior to RRC approval funding has to be committed by the institution and the funding formula between the institution and the program has to be agreed upon. These two factors have come together to make it very difficult to offer pediatric urology fellowships from a funding stand point. Most hospitals value the clinical portion of this training and 88% provide funding for this. By the ABU requiring 2 years of training for the pediatric urology fellow with only one required to be a clinical year faculty members have to provide funding for these fellowship positions for the second, ABU-required year in 24% of the fellowships and in the first year for 4% of the fellowships. Seven academic institutions have the good fortune of having grants or endowments that fund the additional second required year of pediatric urology fellowship.

Pediatric urology fellows are typically board eligible general urologists and as such most medical staffs could admit them to their medical staff and many fellowship programs can and do list their position as a faculty member at the instructor level during the second year. In doing so, services that are offered independently by pediatric urology fellows are eligible to have bills submitted to third party payers and 32% of the fellowships have this additional funding source. At this time, there are no restrictions for how these fellows can bill for independent services they provide. We were unable through this survey instrument to ascertain if these fellows were supervised as a truly educational experience or whether they functioned as a junior faculty or even in a teaching setting for other trainees. Some hospitals have decided against this practice and in our own hospital we have a policy that those who are in training are not eligible to bill independently for services they provide since they are there to learn as part of their fellowship training.

We did not ask about what clinical billings are used for. We did determine that these funds went to support the pediatric urology fellow during institutional or faculty unfunded periods. We did not question whether the fellow-generated funds were sufficient to cover fellowship-related expenses were used for other purposes or were simply added to the general funds of the department or institution.

We also did not survey any other fellowship programs besides pediatric urology approved programs. It would be interesting to determine how other pediatric surgical fellowship and medical fellowships fund their positions but this was not in the scope of this study. Another comparison would be fellowships within urology. Those programs are associated with academic institutions or specialty hospitals such as cancer hospitals and care for Medicare patients allowing for a different funding stream as has been previously described.

With funding of fellowship positions under local control, it is obvious that the “playing field” is not even for the programs and for the fellow’s educational endeavors. Some have questioned the ethics of institutions offering fellowship positions and not having them fully funded by the institutions that benefit from the educational efforts of the training program. There is no way of determining whether the type of funding affects the educational value to the individual fellows or whether it affects the fellow’s inclination toward academic productivity.

Our children’s hospital has chosen to fund the salary only portion of all fellowship trainees including pediatric urology fellowship position for the clinical year required. Benefits must be paid for from other sources. The second year of either clinical or non-clinical/research activities will not be funded by our children’s hospital.

## Conclusion

All but three of the pediatric urology fellowship positions are funded by the institution at which the fellow works for the required clinical year of training. Funding for the second, ABU-required l year is provided by the hospital in 44% of fellowships. In 24%, the faculty members pay for the pediatric urology fellowship out of their clinical income. Only 32% of pediatric urology fellows have bills submitted for their independent services to augment fellowship funding.

## Conflict of Interest Statement

The authors declare that the research was conducted in the absence of any commercial or financial relationships that could be construed as a potential conflict of interest.

## Abbreviations

ACGME, accreditation council for graduate medical education; RRC, residency review committee; CMS, Center for Medicare and Medicaid Services; ABU, American board of urology.
